# Southern Medical College

**Published:** 1879-04-20

**Authors:** 


### *SOUTHERN MEDICAL COLLEGE.*

Having promised to keep our readers posted in regard to this new Institution, we will state that the Building Committee have secured an eligible lot in the most desirable and central portion of the city. Arrangements are already in progress for the erection of a large, elegant, and suitable building, which will be completed and equipped with all the modern and improved facilities for the successful and thorough teaching of medicine in all its departments, on or before the first of October next. Now that the necessary preliminary steps have been taken, the Trustees will at an early day fill the several chairs with teachers selected with reference to their ability and fitness for their respective positions. It is designed to fill the leading Professorships with men of experience and ability, and the special chairs with youug men of brilliant and aspiring minds.

The Institution will take high ground from the beginning, and will be ready to co-operate with the foremost in elevating the standard of Medical education, and in removing the stigma which has been brought upon the profession by the "legal manufacturing of imperfectly educated medical graduates."

